# The influence of physical exercise on the relation between the phase of cardiac cycle and shooting accuracy in biathlon

**DOI:** 10.1080/17461391.2018.1535626

**Published:** 2018-10-26

**Authors:** Germano Gallicchio, Thomas Finkenzeller, Gerold Sattlecker, Stefan Lindinger, Kerstin Hoedlmoser

**Affiliations:** 1 Centre for Cognitive Neuroscience, University of Salzburg, Salzburg, Austria; 2 Department of Sport Science & Kinesiology, University of Salzburg, Salzburg, Austria

**Keywords:** Biathlon, cardiac cycle, electrocardiogram, physical exercise, shooting accuracy

## Abstract

This study examined the influence of physical exercise on the relation between shooting accuracy and the phase of the cardiac cycle in which the shot is fired. Thirteen experienced biathletes (8 females, mean age 17 years) fired from the standing position at rest and right after a submaximal exercise on a bicycle ergometer. Shooting accuracy and the timing of each shot relative to the R-waves of the electrocardiogram (ECG) were recorded. Best shots (with greatest accuracy) and worst shots (with lowest accuracy) were fired prevalently in different phases of the cardiac cycle. In the rest condition, best shots were fired less frequently from 200 to 300 ms and more frequently from 500 to 600 ms after the R-wave, compared to worst shots. In the exercise condition, best shots were fired less frequently from 100 to 200 ms after the R-wave and from 20% to 30% of the R-R interval, compared to worst shots. These findings support the hypothesis that shooting accuracy is influenced by the cardiac cycle phase due to the ballistocardiac recoil generated at each heartbeat. To achieve best results athletes could be trained (e.g. through biofeedback) to fire within a specific phase of the cardiac cycle.

## Highlights

- The phase of the cardiac cycle in which the shot is fired can affect shooting accuracy.
- To shoot accurately, marksmen should avoid firing at a time corresponding with the pressure pulse wave peak.
- Acute physical exercise augments the destabilizing effect of cardiac contraction on shooting accuracy.

## Introduction

Biathlon is an Olympic winter sport that combines cross-country skiing and rifle shooting. Depending on the race format, biathletes complete three to five laps of cross country skiing, covering a distance of 6–20 km while carrying on their back a small-bore rifle with minimum weight of 3.5 kg. Between each skiing lap biathletes complete a shooting session in which they attempt to hit five targets placed at a distance of 50 m, firing from the either the prone or the standing position. A penalty time or penalty skiing distance is given for each target miss. The athlete with the shortest overall time wins the race (International Biathlon Union, 2016).

Research in biathlon provides with the peculiar opportunity to study precision shooting when fine motor control is challenged by the physiological adaptations to intense whole-body physical exercise (Lakie, 2010). On the one hand, successful rifle shooting requires stable rifle hold and posture (Era, Konttinen, Mehto, Saarela, & Lyytinen, 1996; Ihalainen, Kuitunen, Mononen, & Linnamo, 2016, 2018; Sattlecker, Buchecker, Müller, & Lindinger, 2014, 2017). Indeed, at a distance of 50 m even a tiny deflection of the barrel from the ideal alignment translates to a much larger error on the target. On the other hand, biathletes exercise at very high physical loads, with heart rates (HR) of 90% of their maximal capacity while skiing, and 85%–87% when approaching the firing line (Hoffman & Street, 1992). Crucially, intense physical exercise is known to affect postural balance (Paillard, 2012), especially when standing and holding an additional weight such as the rifle (Bermejo et al., 2015).

In order to fire in a moment of postural stillness, marksmen are often trained to shoot in the interval between consecutive heartbeats. Indeed, a series of studies has provided evidence that, when firing from the standing position, the timing of the shot within the cardiac cycle can influence the accuracy of the shot (Helin, Sihvonen, & Hänninen, 1987; Konttinen, Mets, Lyytinen, & Paananen, 2003; Mets, Konttinen, & Lyytinen, 2007).

In a seminal study, Helin et al. (1987) examined the effects of shooting during cardiac systole or diastole on gun stability and shooting accuracy. The authors recorded shooting accuracy, ECG, and gun displacement from six elite rifle and pistol shooters (national champions with 5–30 years of shooting training) and three novice rifle shooters (with 1–2 years of shooting training), as they fired 20 shots under simulated competition conditions. Helin et al. found two lines of evidence that shooting during cardiac systole is associated with worse accuracy than shooting during diastole. First, elite athletes fired almost consistently during diastole, whereas novices fired equally during diastole and systole. Second, novices achieved greater shooting accuracy when they fired during diastole instead of systole. Crucially, by comparing the waveforms of ECG and gun displacement, Helin et al. observed that the gun was momentarily destabilized about 200 ms after the occurrence of an R-wave of the ECG, that is when systolic blood pressure was expected to reach its peak. Helin et al. concluded that gun destabilization was likely to account for the negative effect of shooting during cardiac systole.

More recently, Konttinen et al. (2003) developed a method to examine the prevalence of shots across different phases of the cardiac cycle. They recorded the ECG from 20 participants with little shooting experience (8–11 months of compulsory military service) as they fired 200 shots to a 10-m distant shooting target. Konttinen et al. measured the temporal interval between each shot and the preceding R-wave and expressed such interval as percentage of the R-R interval at the moment of shooting. They found that these participants fired more often in the 10%–50% and less often in the 50%–90% interval of the R-R cycle, compared to uniform distribution of shots across the cardiac cycle. In addition, shots fired in the 50%–70% interval were associated with worse accuracy compared to shots fired in other percentage bins.

Finally, Mets et al. (2007) applied the time course method developed by Konttinen et al. (2003) to study the relation between cardiac cycle and shooting accuracy. They recorded the ECG from 20 experienced adolescent shooters (2–10 years of shooting training) as they fired 200 shots to a 10-m distant shooting target. They identified best and worst shots of each participant using a median split and compared their prevalence in each interval of the cardiac cycle. They found no difference for best and worst shots; however, overall shots were fired more often in the 10%–15% and less often in the 45%–60% interval of the R-R cycle, compared to uniform distribution.

Taken together, these three studies provide evidence that the accuracy of a shot can be influenced by the time within the cardiac cycle when the shot is fired and that this phenomenon may be partly attributed to ballistocardiac recoil interfering with a stable gun hold. However, it is worth to point out that there is little agreement across these studies on what phase of the cardiac cycle provides the optimal timing for accurate shooting. This inconsistency can be attributed to the examination of the lag between the R-wave and the shot as either absolute time (i.e. in ms) or as relative to the R-R interval (i.e. in percentage). While the former can be interpreted in light of ballistocardiac phenomena known to happen at specific times after the R-wave (Cokkinos et al., 1976; Lance & Spodick, 1976), the latter is susceptible to distortions consequent to changes in HR. For example, increases in HR (i.e. shorter R-R cycles) have a greater impact on diastolic than systolic durations (Folkow & Neil, 1971): treating each percentage bin equally may misrepresent the relation between HR changes and the occurrence of cardiac events. The simultaneous examination of R-shot intervals as absolute and relative measure should shed light on the utility of each metric and ensure comparability with existing research regardless of the approach chosen.

To date, the relation between cardiac cycle and rifle shooting has been examined under conditions of rest, but little is known when shots are fired under intense physical load, as in biathlon shooting. Cardiac adjustments to intense physical exercise include shortening of the cardiac cycle and increase in myocardial contractility and blood pressure (Obrist, 1981). Because the effects of the phase of the cardiac cycle on shooting accuracy are likely due to the contraction of the cardiac muscle (Helin et al., 1987) and due to the fact that intense physical exercise influences the cardiac cycle (Obrist, 1981), physical exercise may influence the relation between cardiac cycle and shooting accuracy.

The aim of this study was to explore the influence of intense physical exercise on the relation between the phase of the cardiac cycle and shooting accuracy. We conducted a series of novel analyses on ECG and performance data collected from experienced adolescent biathletes as they fired under conditions of rest and intense physical exercise. This dataset further included neurophysiological recordings and analyses (cf. Gallicchio, Finkenzeller, Sattlecker, Lindinger, & Hoedlmoser, 2016). We tested two hypotheses. First, that the count of best shots (with highest accuracy) and worst shots (with lowest accuracy) differed as a function of the phase of the cardiac cycle. Second, that worst shots were prevalent over best shots at a time corresponding with the estimated pressure pulse wave peak. Namely, we expected that this interval occurred around 200 ms after the R-wave at rest (Helin et al., 1987) and earlier when shots were fired after physical exercise, due to the shortening of the diastolic and systolic phases of the cardiac cycle (Obrist, 1981).

## Methods

### Participants

Thirteen experienced adolescent biathletes (5 males, 8 females; age: *M* = 17.08, *SD* = 1.66 years; BMI: *M* = 20.72, *SD* = 1.25 kg/m^2^) from the Federal youth teams of Styria and Salzburg participated in this study. Participants reported a biathlon experience of 5–8 years and had a HR-max of 200.39 bpm (*SD* = 6.36), measured within the seven months preceding testing through spiroergometry.

### Procedure

Each participant was tested individually in an indoor range with controlled wind and temperature conditions. After a 10-min shooting practice and rifle zeroing, participants were instrumented for physiological recording and then completed the shooting task. Rifle zeroing indicates the adjustment of the rifle scope, so that the sight was aligned with the trajectory of the bullet towards the target. The task consisted in shooting at rest (i.e. no additional physical load) and after intense physical exercise. The study was conducted in respect of the Code of Ethics of the World Medical Association (Declaration of Helsinki) and was approved by the local ethics committee.

### Physiological recording

Bipolar ECG was recorded in the chest-configuration lead-II montage and digitized with a sampling rate of 1024 Hz (eegosport system; ANT Neuro, Netherlands). R-waves of the ECG were identified using QRSTool (Allen, Chambers, & Towers, 2007) and their timings were imported into MATLAB (MathWorks, MA) for further processing. Shots were detected by a microphone interfaced with the recording system via a stimulus box (StimTracker, Cedrus, CA). During exercise, HR was monitored by a chest belt (Polar Electro, Finland) and fed back to the participants to make sure they exercised at the required intensity of 90% of maximal HR.

### Shooting task

Participants fired from the standing position at a shooting target (diameter of 11.5 cm) placed at a distance of 50 m, using their personal rifle and ammunition. Each participant fired 120 shots, divided in 24 blocks of each 5 consecutive shots. For the first 12 blocks (rest condition) participants fired under no physical load. Each of these blocks was separated by a break of approximately 2 min. For each of the second 12 blocks (exercise condition) participants completed a 3-min bicycle ergometer exercise at 90% of their maximal HR prior to firing 5 consecutive shots. The intensity of the physical load was based on observations of elite biathletes in competition (Hoffman & Street, 1992). Participants were instructed to shoot as accurately as possible in their individual competition pace (i.e. under time pressure) in order to properly simulate race conditions. No feedback was given about the accuracy of each shot.

### Measures

**Figure 1.**
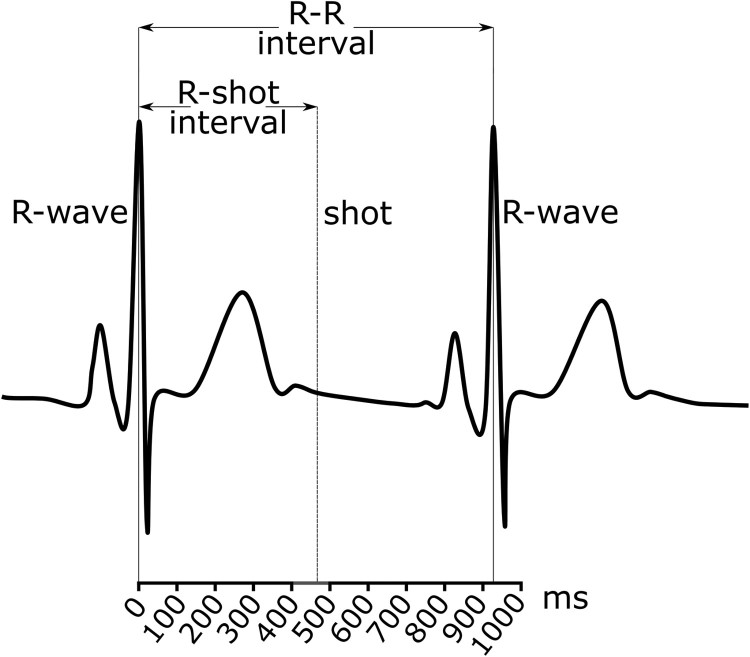
R-shot_ms_ times were clustered in interval bins of 100 ms. In this example, the shot was fired between 400 and 500 ms from the occurrence of the R-wave that preceded that shot.

Shooting accuracy was recorded by a computer-controlled device (SA 921, SiusAscor, Switzerland) and measured in target rings, with values ranging from 0 (outside the outer ring) to 10.9 (centre of the target). The timings of the R-waves of the ECG and of the shots were combined to compute R-R and R-shot intervals (Figure 1). The R-R interval was computed as the time (ms) between the two R-waves that preceded and followed a shot. The R-shot interval was computed as the time (ms) between the R-wave that preceded a shot and the shot itself. In addition, a percentage measure of the R-shot interval was computed relative to the R-R interval (i.e. R-shot interval in ms divided by R-R interval in ms, multiplied by 100; Konttinen et al., 2003; Mets et al., 2007). Hereafter “R-shot_ms_” and “R-shot_%_” will be used to refer to the two types of R-shot intervals computed in this study. The examination of both measures was conducted to permit comparison between the two approaches and with all relevant studies published to date. A total of 28 shots (on average 1.07 shots per participant / condition) were excluded from further analyses due to uncertainty in the measurement of shooting accuracy or presence of artefacts in the ECG signal, leaving a minimum of 50 shots per each participant in each of the two conditions.

### Design and statistical analyses

A regression analysis was conducted to test the overarching research question asking whether physical exercise moderated the relation between the phase of the cardiac cycle and shooting accuracy. The regression included shooting accuracy as continuous outcome, quadratic “R-shot_ms_” as continuous predictor, and “Load” (0 = rest, 1 = exercise) as categorical predictor (i.e. moderator). A significant Load × R-shot_ms_ interaction was taken to indicate that physical exercise moderated the relation between cardiac cycle and shooting accuracy. We employed a mixed-effect model on trial-level data to account for the repeated-measure nature of the design, using the nlme package for R (Pinheiro, Bates, DebRoy, & Sarkar, 2018). The interested reader can access the full specifications of the regression model in the supplemental online material.

Shooting accuracy, R-R, R-shot_ms_, and R-shot_%_ intervals were each analyzed as a function of “Load” (rest, exercise) and “Performance” (best, worst) through two-way repeated-measure ANOVAs. “Performance” identified the 15 best and the 15 worst shots, defined as the shots with respectively highest and lowest accuracy fired by each participant in each load condition (cf. Mets et al., 2007). This criterion was preferred over hit-miss distinction because it allowed to focus on the lowest and highest tails of the performance spectrum while promoting signal-to-noise ratio stability (i.e. an identical number of trials was employed for the factors “Load” and “Performance”).

To evaluate the prevalence of best and worst shots within different phases of the cardiac cycle, we split the R-R interval into several bins. The factor “R-shot_ms_ bins” identified 100-ms bins within which the R-shot_ms_ intervals were sorted (cf. Flynn & Clemens, 1988). Because no shots were fired 1200 and 600 ms after the R-wave, respectively for the rest and exercise conditions, this factor identified 12 bins for the rest condition (i.e. 0–100, 100–200, up to 1100–1200 ms) and 6 bins for the exercise condition (i.e. 0–100, 100–200, up to 500–600 ms). The factor “R-shot_%_ bins” identified ten percentage bins (i.e. 0%–10%, 10%–20%, until 90%–100%) within which the R-shot_%_ intervals were sorted (cf. Konttinen et al., 2003; Mets et al., 2007). The count of shots was analyzed as a function of “Performance” (best, worst) and either “R-shot_ms_ bins” or “R-shot_%_ bins” through repeated-measure ANOVAs. These ANOVAs were conducted separately per each condition (i.e. rest and exercise). An interaction between “Performance” and either “R-shot_ms_ bins” or “R-shot_%_ bins” was taken to indicate that best and worst shots were fired with different prevalence across the cardiac cycle (hypothesis 1); therefore, only two-way interactions were examined. Significant interactions were further examined with paired-sample t-tests to evaluate in which phases of the cardiac cycle the count of best and worst shots differed (hypotheses 2). Huynh-Feldt correction was applied to the results of the repeated-measure ANOVAs when Mauchly’s test (*p* < .05) revealed that the assumption of sphericity was violated (Huynh & Feldt, 1970). When this correction was applied, the original degrees of freedom and the associated ϵ were reported. Partial eta-squared (η_p_^2^) was reported as measure of effect size: values of .02, .13, and .26 were taken to reflect small, medium, and large effects respectively (Cohen, 1992). Statistical analyses were conducted using IBM SPSS v.24.0.

## Results

The regression analysis yielded a significant R-shot_ms_ × Load interaction (*b* = 0.000004, *t*(1526) = 2.32, *p* = .02) indicating that the relation between the phase of the cardiac cycle and shooting accuracy was more pronounced in the exercise than in the rest condition. No simple effect emerged for quadratic R-shot_ms_ (*b* = 0.00, *t*(1516) = 0.38, *p* = .70) or Load (*b* = −0.34, *t*(1516) = −1.77, *p* = .08).

The results of Load × Performance ANOVAs on shooting accuracy, R-R, R-shot_ms_, and R-shot_%_ intervals are showed in Table I. The R-R interval decreased from 846.46 ms of the rest condition to 389.76 ms of the exercise condition. These values correspond to HR of 70.88 and 153.94 bpm, for the rest and exercise conditions respectively. The R-shot_ms_ interval decreased from rest to exercise, similarly to the R-R interval. Shooting accuracy did not change from rest to exercise and was greater for best than worst shots. The R-R interval did not differ for best and worst shots. However, R-shot_ms_ and R-shot_%_ intervals were longer for best compared to worst shots, indicating that, independently of the load condition, shots with highest accuracy were fired later in the cardiac cycle than shots with lowest accuracy.

**Table I.** Mean (± standard deviation) of shooting accuracy, R-R interval, R-shot interval, and relative R-shot interval, as a function of “Load” (rest, exercise) and “Performance” (best, worst), with the results of repeated-measure ANOVAs.

	Rest		Exercise		Load		Performance		Load × Performance	
Measures	Best	Worst	Best	Worst	*F*_1,12_	*η_p_*^2^	*F*_1,12_	*η_p_*^2^	*F*_1,12_	*η_p_*^2^
Shooting accuracy (0.0–10.9)	8.1 ± 0.8	1.1 ± 1.2	7.9 ± 0.9	1.3 ± 1.3	0.00	.000	1114.59**	**.989**	0.82	.064
R-R interval (ms)	841.4 ± 173.2	851.5 ± 160.5	391.4 ± 65.3	388.1 ± 59.6	154.13**	**.928**	0.20	.016	0.91	.070
R-shot_ms_ interval (ms)	431.5 ± 89.0	397.4 ± 85.8	205.2 ± 44.5	180.0 ± 27.1	105.08**	**.898**	8.03*	**.401**	0.17	.014
R-shot_%_ interval (%)	51.3 ± 6.0	46.3 ± 5.0	52.8 ± 8.7	46.9 ± 7.2	0.44	.035	9.04*	**.430**	0.04	.003

*P ≤ .05.

**P < .001.

**Figure 2.**
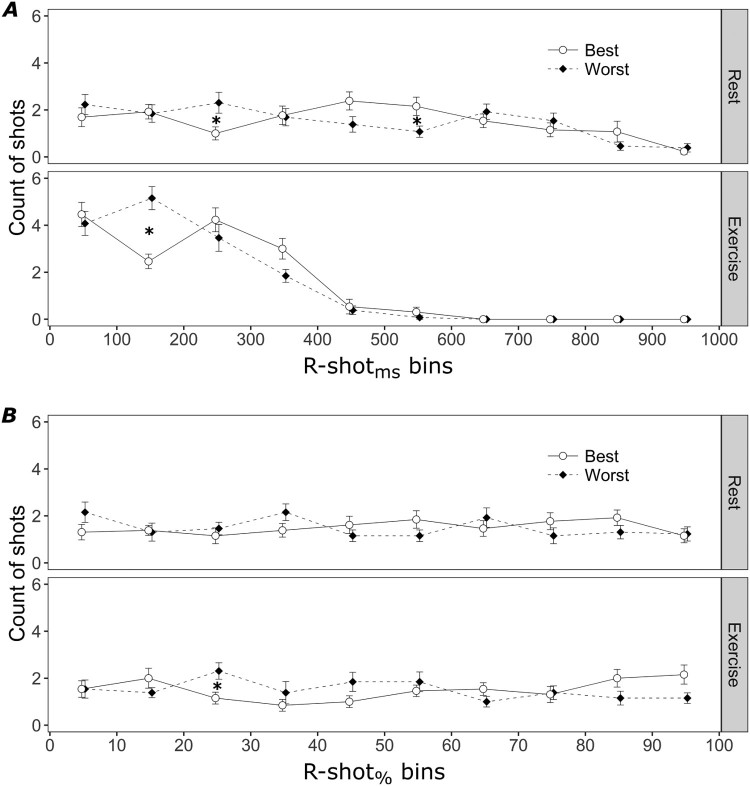
Count of best and worst shots as a function of Performance and either R-shot_ms_ bins (panel A) or R-shot_%_ Bins (panel B), for the rest and exercise conditions. Error bars indicate the *SE* of the means.

The Performance × R-shot_ms_ bins ANOVAs conducted on the count of shots (Figure 2A) yielded interaction terms that were significant for both conditions: rest, *F*_11,132_ = 2.19, *P* = .04, ϵ = .624, *η_p_*^2^ = .155; exercise, *F*_5,60_ = 4.92, *P* = .009, ϵ = .508, *η_p_*^2^ = .291. T-tests conducted on the interaction for the rest condition indicated that, compared to worst shots, best shots were fired less frequently in the 200–300 ms bin, *t*_12_ = 2.50, *P* = .03, and more frequently in the 500–600 ms bin, *t*_12_ = 3.27, *P* = .007. T-tests conducted on the interaction for the exercise condition indicated that best shots were fired less frequently than worst shots in the 100–200 ms bin, *t*_12_ = 5.55, *P* < .001.

The Performance × R-shot_%_ bins ANOVAs conducted on the count of shots (Figure 2B) yielded interaction effects that were non-significant for the rest condition, *F*_9,108_ = 1.40, *P* = .20, *η_p_*^2^ = .105, and significant for the exercise condition, *F*_9,108_ = 2.13, *P* = .03, *η_p_*^2^ = .151. T-tests conducted on the interaction for the exercise condition indicated that best shots were fired less frequently than worst shots in the 20%–30% of the R-R bin, *t*_12_ = 2.74, *P* = .02. Based on mean R-R interval, this percentage bin corresponds with a range of 77.95–116.93 ms from the occurrence of the R-wave.

## Discussion

This study explored the relation between shooting accuracy and the time within the cardiac cycle in which the shot is fired, at rest and under intense physical load. In line with our hypotheses we found that (a) best and worst shots were fired prevalently in different phases of the cardiac cycle and that (b) physical exercise influenced the relation between the phase of the cardiac cycle and shooting accuracy.

Physical exercise did not impair shooting accuracy (cf. Hoffman, Gilson, Westenburg, & Spencer, 1992; Luchsinger, Sandbakk, Schubert, Ettema, & Baumeister, 2016; Vickers & Williams, 2007); however, it influenced the relation between shooting accuracy and the cardiac cycle. First, the regression analysis revealed that physical exercise moderated the relation between shooting accuracy and the phase of the cardiac cycle: this relation was stronger for the exercise compared to the rest condition. Second, the Load × Performance ANOVAs indicated that the R-R and the R-shot_ms_ intervals shortened significantly from the rest to the exercise condition. These effects were highly expected based on the increase in HR that accompanies physical exercise (Obrist, 1981). Third, the Performance × R-shot_ms_ ANOVAs revealed that best shots were associated with longer R-shot_ms_ intervals than worst shots. This result indicates that, irrespectively of load conditions, best shots were fired later than worst shots within the cardiac cycle. This finding is consistent with previous observations that firing later in the cardiac cycle is associated with expertise and greater accuracy (Helin et al., 1987).

To examine the relation between the phase of the cardiac cycle and shooting accuracy, we sorted R-shot intervals into 100-ms bins from to the occurrence of the R-wave (R-shot_ms_ bins; cf. Flynn & Clemens, 1988) and into percentage bins relative to the duration of the R-R interval (R-shot_%_ bins; cf. Konttinen et al., 2003; Mets et al., 2007). The analyses on the prevalence of best and worst shots within both types of bins confirmed that shooting accuracy was associated with the phase of the cardiac cycle in which the shot is fired (Figure 2). When shooting at rest, best shots were fired less frequently from 200 to 300 ms and more frequently from 500 to 600 ms after the R-wave, compared to worst shots. When shooting after physical exercise, best shots were fired less frequently from 100 to 200 ms after the R-wave and from 20% to 30% of the R-R interval, compared to worst shots.

These findings provide indirect support to the hypothesis that the relation between cardiac cycle and shooting accuracy is attributable to the ballistocardiac effect (Helin et al., 1987). First, the interval from 200 to 300 ms after the R-wave, in which worst shots were fired more frequently than best shots at rest, corresponds with the time when most individuals report feeling the sensation of their heartbeat. For example, Brener and Kluvitse (1988) found that most individuals perceive their heartbeats in the 200–300 ms interval after the R-wave. Other studies examining cardiac interoception have reported similar intervals – i.e. 200–400 ms (Yates, Jones, Marie, & Hogben, 1985) and 150–350 ms (Ring & Brener, 1992). These studies also revealed that the sensation of the heartbeat is localized to several parts of the body including the chest, the abdomen, and the hands (e.g. Brener & Kluvitse, 1988). The timing and location of the heartbeat sensation are compatible with the account that, within those times, individuals perceive the vibrations generated from the pressure pulse wave travelling through the body. Second, the fact that best shots were prevalent over worst shots from 500 to 600 ms after the R-wave, when shooting at rest, can be interpreted as a by-product of the ballistocardiac effect. Namely, it could be speculated that this interval represents the time where the negative effect of the ballistocardiac recoil on the destabilization of the rifle is at its minimum. Finally, the intervals where worst shots are prevalent over best shots – i.e. 200–300 and 100–200 ms for the rest and the exercise conditions, respectively – can be associated with the ejection of blood from the left ventricle. By using data from previous literature (Cokkinos et al., 1976; Lance & Spodick, 1976; Mertens, et al., 1981) we estimated^1^ that the ejection of blood from the left ventricle would have occurred from 107.16 to 358.58 ms and from 46.14 to 232.41 ms after the occurrence of the R-wave, respectively for the rest and exercise conditions. These latencies include the bins described above and could represent the timing of the jerk generated by the contraction of the left ventricle.

To date this is the only study evaluating the utility of two different approaches to examine R-shot intervals as absolute measure in ms (cf. Flynn & Clemens, 1988) or percentage measure relative to the duration of the cardiac cycle (cf. Konttinen et al., 2003; Mets et al., 2007). The separate analyses of absolute and percentage bins yielded similar results for the exercise condition but not for the rest condition. Namely, performance effects at rest emerged for absolute bins but not for percentage bins. The inconsistent findings between the two approaches may be explained on the basis of their different advantages. On the one hand, absolute intervals can reflect, albeit indirectly, the occurrence of specific cardiac events, such as left ventricular ejection. On the other hand, percentage intervals allow to control for variations of the R-R interval; however, they can also smear transient effects across multiple bins due to the fact that the ratio between systole and diastole is not constant and depends on the duration of the cardiac cycle. For instance, a reduction of the R-R interval corresponds to a much greater reduction in the diastolic than in the systolic phase (Folkow & Neil, 1971, p. 185).

### Limitations and future directions

This study revealed important associations between shooting accuracy and the phase of the cardiac cycle. However, these findings need to be considered in light of the following limitations. First, our results do not imply a causal relationship between shooting in a certain phase of the cardiac cycle and the accuracy of the shot. This is due to the fact that we could not manipulate experimentally the timing of the shot in the different phases of the cardiac cycle. Second, the examination of the timing of the R-waves of the ECG does not provide a direct link with cardiac events except the depolarization of the left ventricle. Therefore, other relevant cardiac events, such as the ejection of blood and the ballistocardiac recoil, had to be inferred via indirect procedures. Mechanistic studies interested in a more direct test should consider complementing ECG recordings with further measurements, such as the destabilization of the rifle (Helin et al., 1987) and impedance cardiography (Sherwood et al., 1990). Third, although supporting a ballistocardiac account, the findings of this study do not rule out that other factors may contribute to explaining the relation between cardiac cycle and shooting accuracy. For example, changes in brain activity induced by cardiac events such as the heartbeat (Lechinger, Heib, Gruber, Schabus, & Klimesch, 2015; Schandry, Sparrer, & Weitkunat, 1986) may influence cognitive processes (e.g. motor control) responsible for fine visuomotor performance. Finally, the timing within the cardiac cycle can only explain a minimal portion of the shooting result: as revealed by Figure 2, best and worst shots were fired across the whole cardiac cycle. Accordingly, other factors such as aiming accuracy, rifle stability, cleanness of triggering (Ihalainen et al., 2016), focus of attention, and expertise (Doppelmayr, Finkenzeller, & Sauseng, 2008)—which are independent of the cardiac cycle phenomenon—can influence shooting accuracy.

## Conclusions

This study examined the relation between shooting accuracy and the phase of the cardiac cycle in biathlon shooting under conditions of rest and physical exercise. The findings of this study provide indirect support to a ballistocardiac account of the relation between cardiac cycle and shooting accuracy: shots with greatest accuracy are fired away from a phase of the cardiac cycle that is compatible with the pressure pulse wave peak occurring after a heartbeat. Importantly, this study revealed for the first time that physical exercise influences the destabilizing effect of cardiac contraction on shooting accuracy by augmenting it and making it occur earlier with the cardiac cycle.

This study suggests that athletes could improve their shooting performance by firing in a specific time of the cardiac cycle that is immune from the destabilizing effects of heartbeat jerking on gun hold. These findings open interesting avenues for future applications whereby marksmen could be trained to shoot within a phase of the cardiac cycle that is associated with greater performance. This training could include biofeedback of cardiac activity or rifle destabilization and should prove particularly helpful to athletes performing under conditions of intense physical exercise.

## Supplementary Material

Supplemental Material

## Note

1. Systolic time intervals, i.e., pre-ejection period (PEP) and left ventricular ejection time (LVET) were estimated in the following way. LVET was estimated as a function of Heart Rate (HR) using regression equations for (a) rest LVET = 379–1.8 × HR ± 11.0 and (b) dynamic exercise LVET = 371–1.2 × HR ± 13.9 (Lance & Spodick, 1976). PEP was estimated for (a) rest using average durations of 107.16 ± 1.61 ms computed from 26 healthy individuals (Cokkinos et al., 1976) and (b) dynamic exercise as a function of Heart Rate (HR) using the regression equation PEP = 138.5–0.60 × HR ± 25.5 ms (Mertens et al., 1981). It is important to note that at rest PEP is considered independent of HR (Cokkinos et al., 1976; Mertens, 1981).
