# Supplementary material for: The influence of physical exercise on the relation between the phase of cardiac cycle and shooting accuracy in biathlon
Source: Eur J Sport Sci. 2018 Oct 26;19(5):567–75. doi: 10.1080/17461391.2018.1535626 (PMC6518456; doi:10.1080/17461391.2018.1535626)
Supplement: Supplemental Material [file TEJS_A_1535626_SM6913.docx]

**Appendix: Regression analysis**

A mixed-effect regression model was evaluated to test the hypothesis that physical exercise moderated the relation between shooting accuracy and the phase of the cardiac cycle. This model was fit by maximum likelihood using the ‘nlme’ package for the R statistical software (Pinheiro et al., 2018). Shooting accuracy was entered as trial-level continuous outcome. Quadratic R-shot_ms_ interval was entered as trial-level continuous predictor. Load (rest, exercise) was entered as dummy-coded categorical predictor (0 = rest, 1 = exercise). The interaction between quadratic R-shot_ms_ interval and Load was also entered as predictor. The model was specified so that each participant was allowed his/her own intercept and R-shot interval coefficient. R-shot_ms_ was entered in the quadratic form to account for the non-linear relation between the phase of the cardiac cycle and shooting accuracy. The exact shape of this relation varied across participants; however, a quadratic relation (i.e., U shape) appeared the best fit (**Figure A.1**).


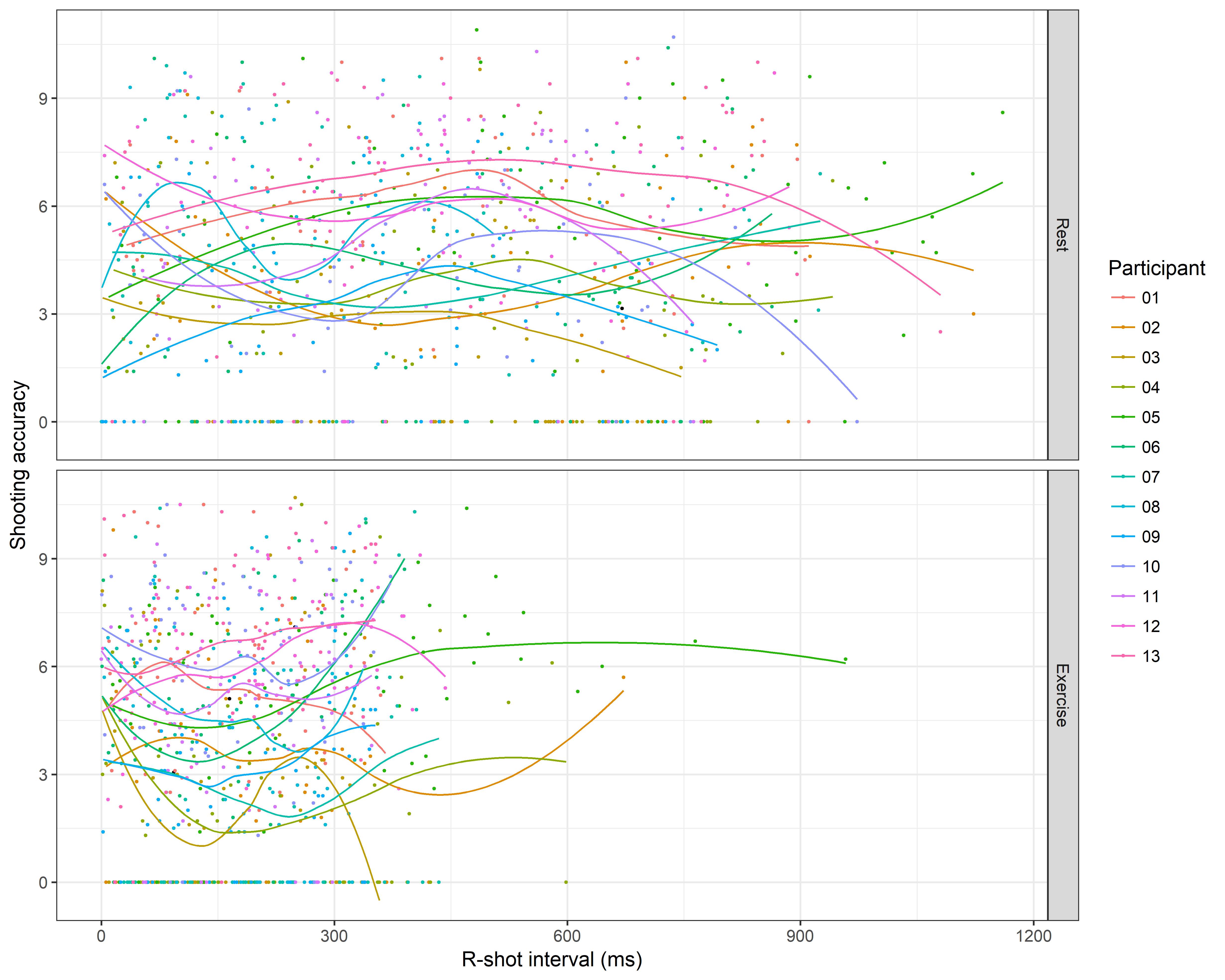


**Figure A.1** Scatterplot of shooting accuracy as a function of R-shot interval, separately for the rest and exercise conditions. Each dot represents a trial (i.e., shot) and smooth lines represent the best non-linear fit, separately per each subject. Each participant is colour-coded.
